# Measurement of total factor productivity of green agriculture in China: Analysis of the regional differences based on China

**DOI:** 10.1371/journal.pone.0257239

**Published:** 2021-09-10

**Authors:** Shen Zhong, Yuexin Li, Jian Li, Huiying Yang

**Affiliations:** 1 Northeast Agricultural University, Harbin, Heilongjiang, PR China; 2 School of Finance, Harbin University of Commerce, Harbin, Heilongjiang, PR China; 3 School of Economics, Harbin University of Commerce, Harbin, Heilongjiang, PR China; Shenzhen University, CHINA

## Abstract

China’s agricultural economy is developing rapidly, but the unbalanced regional development is still a key issue that needs to be discussed today. By studying the total factor productivity of green agriculture and its factors, this paper analyzes the regional differences in time and space changes between the eastern, central and western parts of China. In this paper, the total factor productivity of green agriculture is calculated and decomposed by Metafrontier Malmquist-Luenberger index based on directional distance function. The results are as follows: First, the total factor productivity level of green agriculture in China is increasing year by year, but the overall level is still at a low level and has greater volatility; Second, although the total factor productivity of green agriculture shows an upward trend, the three regions show a downward trend in turn, which has great differences; Third, there are obvious differences in technological efficiency, optimal production potential and technological gap between the eastern, central and western regions, and there are great differences in productivity among regions and provinces. Based on the results, this paper puts forward policy recommendations, according to the regional heterogeneity, from a number of angles to rely on the joint efforts of many parties to improve the level of total factor productivity of green agriculture.

## 1. Introduction

Over the past 40 years of reform and opening-up, China’s agriculture has made outstanding contributions to ensuring people’s lives [1,2]. According to the *China Statistical Yearbook in 2020*, the GDP of China’s primary industry has increased from 101.85 billion yuan in 1978 to 7046.67 billion yuan in 2019. It has achieved rapid growth and feeds more than 20% of the whole world’s population, although China occupies less than 10% of the world’s arable land [1,3–5]. However, China’s agricultural development has never got rid of the production mode of high yield and high consumption [6], which has led to a sharp increase in resource consumption [1] and environmental pollution [7,8]. With the development of economic globalization, the production mode with low efficiency and large monthly pollution will cause global waste of resources and environmental pollution [9,10], and it has important theoretical and practical value to improve the total factor productivity of green agriculture in a comprehensive way. Therefore, it is advisable to measure the total factor productivity of green agriculture [11].

At the present stage, China’s agricultural development is mainly manifested in the following situations: First, there is a large amount of energy waste in China’s agricultural production process [12–15]. Scholars predict that China’s energy consumption in agricultural will meet 161.61 million tons of standard coal equivalent in 2025. That’s twice as much as in 2016 [16]. Second, under the production mode of high yield and high consumption [1], on the one hand, "high consumption" makes excessive waste of input factors lead to the deterioration of agricultural ecological environment [7,8,16] and the formation of external diseconomy [17]; On the other hand, "high output" will be accompanied by "high negative output", and the prevention and control effect is not good to destroy the environment [18–21]. Third, China is vast, and due to the differences in economic development [22–24], factor endowment and natural geological conditions, agricultural development is uneven [25], which also brings challenges to the future development of agriculture in China [26–28]. Therefore, when calculating the total factor productivity of green agriculture, the selection of input and output indicators is very important [29,30].

Based on this, this paper makes a reasonable measurement of total factor productivity of green agriculture, considering the spatial and temporal differences between the eastern, central and western regions.

## 2. Literature review

As far as the calculation method and model of agricultural total factor productivity is concerned, data envelope analysis (DEA) and stochastic frontier analysis (SFA) are applied to the calculation of agricultural total factor productivity by most scholars. Stochastic frontier model (SFA), as the representative of the parameter method [31,32], the boundary constructed by SFA conforms to the characteristics of agricultural production [33], but it needs to set specific production functions in advance. Data Envelopment Analysis (DEA), as the representative of nonparametric method [34], used linear programming to treat the same type of decision making unit DMU (Decision Making Units) according to multi-input index and multi-output index. It doesn’t have to preset function form [34,35]. So this paper chooses DEA to do further calculation.

In the subsequent empirical study, Chung et al. [36] pioneered the combination of directional distance function and ML (Malmquist-Luenberger) productivity index to consider the impact of the agricultural total factor productivity based on environmental pollution. With the deepening of the research, scholars have found some limitations of the traditional ML index [37,38], and have established the expansion form. Compared with the traditional ML index, Metafrontier Malmquist-Luenberger index compensates for the neglect of group heterogeneity. The group heterogeneity is included in the research process, the samples are divided into several groups, and the concepts of common frontier and group frontier are introduced, which is more suitable for regional difference analysis [39,40]. Therefore, Metafrontier Malmquist-Luenberger index based on directional distance function is selected to measure and decompose the green total factor productivity of Chinese agriculture.

As far as the selection of indicators for different environmental factors (unexpected outputs), the view of total factor productivity considering the constraints of resources and environmental pollution has been agreed [41–43], but it is not agreed in the calculations of the total factor productivity with the treatment of environmental factors.

The view represented by Thijssen [44] is that environmental factors are used as input variables, and Hailu [45] also used this method to calculate different industries or regions. This method is feasible in theory, but in the actual production process, it is difficult to maintain the total proportion of environmental pollution and input resources, and it is also difficult to reflect the real agricultural production process, so it is not suitable to deal with agricultural environmental pollution factors according to this method. The view represented by Ball et al. [46], Nanere et al. [47] and Shen et al. [48] is that environmental factors are regarded as unwanted output variables, which means that the result of agricultural production is environmental pollution. Agricultural production has not only expected output of agricultural products, but also non-expected output of non-point source pollution and carbon emissions, which accords with the actual agricultural production process. So this paper regards these two types of environmental pollution elements as unexpected outputs.

In the selection of agricultural pollutants, Fei and Lin [16] used CO_2_ as an unexpected output to measure the comprehensive efficiency of agricultural energy and CO_2_ in China. Wang and Lin [49] and Yang et al. [50] calculated CO_2_ emissions based on IPCC guidelines. Boers [51] pointed out that 60% of total nitrogen and 40% -50% of total phosphorus emitted from surface water in the Netherlands are derived from agriculture. Haregeweyn et al. [52] provided the spatial and temporal variations of soil erosion by the agricultural non-point source pollution model (AGNPS). Li [53] defined the ML productivity index model of agricultural non-point source pollution as a non-desirable output in terms of the "green productivity revolution". It can be seen that the selection of agricultural pollutants in academic circles mostly stays at the level of single carbon emissions or non-point source pollution, lacking comprehensive investigation of the two. Su et al. [54] proposed that “In order to curb the negative impact of agricultural production on the environment and improve the level of sustainable agricultural development, it is necessary to quantify the sustainability of different types of agricultural production”. Therefore, carbon emissions and agricultural non-point source pollution are combined as unexpected outputs into green agriculture. Previous literature research mostly stays on single pollutant accounting, and there are some gaps in the comprehensive calculation of pollutant emissions.

In order to remedy the shortcomings of the existing research, this paper mainly focuses on the following three aspects: First, this paper uses the latest year data of 23 years to speculate the total factor productivity of green agriculture in 30 provinces of China for the first time, which has a large time span and stronger reference. Second, this paper considers the emissions of various pollutants more comprehensively, uses IPCC’s method to calculate carbon emissions, and uses the assessment method of non-point source pollution investigation of unit analysis to calculate non-point source pollution, the accounting method is reasonable, and the result is true. Third, this paper focuses on the regional differences in the eastern, central and western regions of China, and comprehensively examines the dynamic evolution process of the three regions based on the dimension of time and space, which supplements the existing research results and has a certain theoretical and practical significance.

## 3. Methodology

In this paper, we mainly select the panel data of 30 provinces in China from 1997 to 2019, and construct a MML (Metafrontier Malmquist-Luenberger) index model based on directional distance function to measure and decompose the total factor productivity of green agriculture in different regions.

### 3.1. Directional distance function

Assuming that each province is a production decision making unit (DMU), each province in the case of *K* production factors input $x=(x_{1},x_{2},\cdots x_{K})\in R_{+}^{K}$. *M* kinds of expected output $y=(y_{1},y_{2},\cdots y_{M})\in R_{+}^{M}$, and *N* kinds of unexpected output $b=(b_{1},b_{2},\cdots b_{N})\in R_{+}^{N}$ can be obtained. Therefore, the input-output value (*x*^*i*,*t*^,*y*^*i*,*t*^,*b*^*i*,*t*^) in the *t* period of province *i* is constructed as follows:

$$P^{t}(x^{t})=\{(y^{t},b^{t}:x\;can\;produce\;(y^{t},b^{t})\}$$ (1)

When the decision unit production possibility set *P*^*t*^ satisfies the unexpected output to be 0, the expected output will also be 0. If the unexpected output can be disposed of under the condition that the unexpected output can be disposed of, the directional distance function can be defined as:

$${\vec{D}}_{0}(x,y,b;g_{y},g_{\partial })=max\{\beta |(y+\beta g_{y},b-\beta g_{b})\in P(x)\}$$ (2)

*β* is the directional distance function value, *g* = (*g*_*y*_,*g*_*b*_) is the direction vector, generally take *g* = (*y*,−*b*), and the purpose of using the directional separation function is to maximize the expected output (*y*) and minimize the expected output (*b*).

### 3.2. Direction distance function DEA model based on common frontier

**Fig 1:**
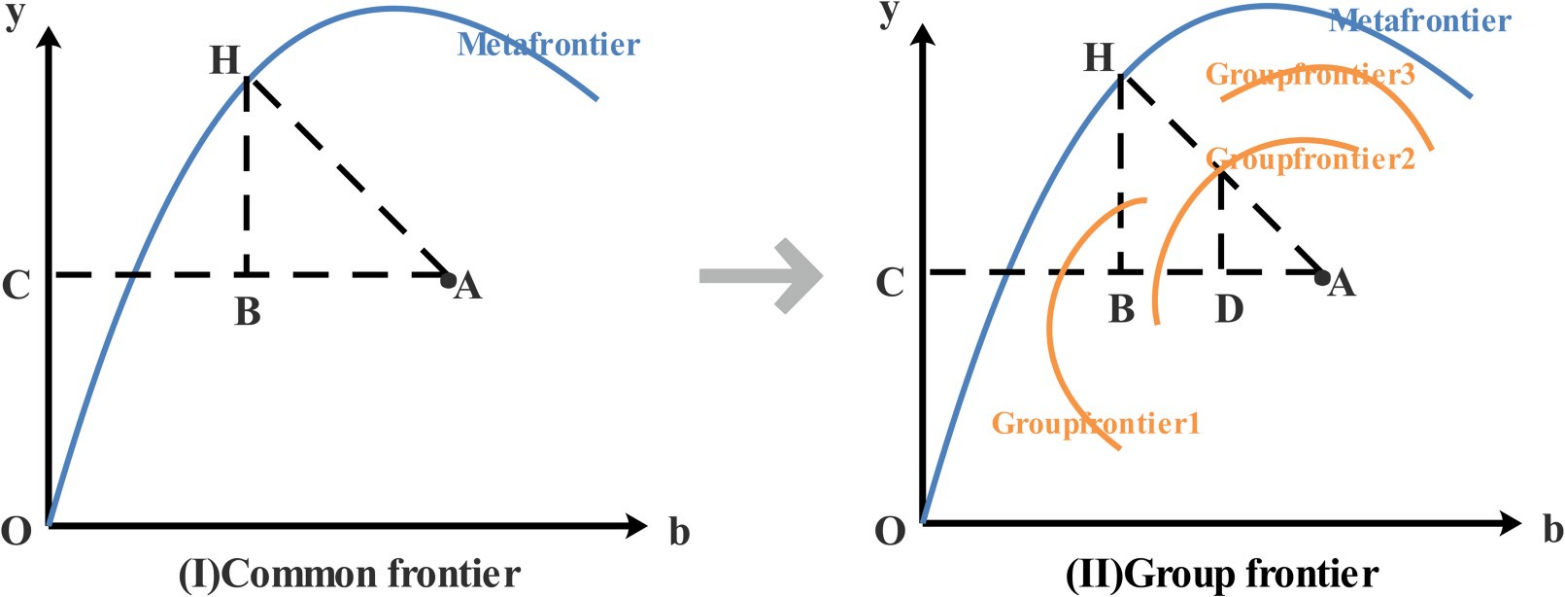
Common frontier and group frontier.

The idea of heterogeneity based on the common frontier analysis method originated from the concept of "Meta-frontier" proposed by Hayami and Ruttan [55], and has been widely used in efficiency measurement. For example, Fig 1 has three group frontiers under the common frontier, which represent the eastern, central and western parts of the country according to geographical regions. The total factor productivity of the DMU at the common frontier and the group frontier can be defined as:

$$\begin{array}{l} 1-\beta^{meta}=\frac{\left\|CB\right\|}{\left\|CA\right\|};\\ 1-\beta^{group}=\frac{\left\|CD\right\|}{\left\|CA\right\|}=\frac{\left\|CB\|+\|BD\right\|}{\left\|CA\right\|} \end{array}$$ (3)

According to Chiu et al. [56], *β*^*meta*^ and *β*^*group*^ can be calculated by the following models:

$$\begin{array}{l} {\vec{D}}_{0}(x^{t},y^{t},b^{t};y^{t},-b^{t})=max\beta^{m}\\ s.t.\left\{ \begin{array}{l} \sum_{t=1}^{T}\sum_{v=1}^{V_{m}}\mu_{v}^{t}x_{vk}^{t}\leq x_{k}^{t},k=1,\ldots ,K\\ \sum_{t=1}^{T}\sum_{v=1}^{V_{m}}\mu_{v}^{t}y_{vm}^{t}\geq (1+\beta^{m})y_{m}^{t},m=1,\ldots ,M\\ \sum_{t=1}^{T}\sum_{v=1}^{V_{m}}\mu_{v}^{t}b_{vn}^{t}=(1-\beta^{m})b_{n}^{t},n=1,\ldots ,N\\ \mu_{v}^{t}\geq 0;v=1,\ldots ,V_{m};t=1,\ldots ,T \end{array}\right. \end{array}$$ (4)

$$\begin{array}{l} {\vec{D}}_{0}(x^{t},y^{t},b^{t};y^{t},-b^{t})=max\beta^{g}\\ s.t.\left\{ \begin{array}{l} \sum_{t=1}^{T}\sum_{v=1}^{V_{g}}\theta_{v}^{t}x_{vk}^{t}\leq x_{k}^{t},k=1,\ldots ,K\\ \sum_{t=1}^{T}\sum_{v=1}^{V_{g}}\theta_{v}^{t}y_{vm}^{t}\geq (1+\beta^{g})y_{m}^{t},m=1,\ldots ,M\\ \sum_{t=1}^{T}\sum_{v=1}^{V_{g}}\theta_{v}^{t}b_{vn}^{t}=(1-\beta^{g})b_{n}^{t},n=1,\ldots ,N\\ \theta_{v}^{t}\geq 0;v=1,\ldots ,V_{g};t=1,\ldots ,T \end{array}\right. \end{array}$$ (5)

Among them, *K*, *M* and *N* are the quantities of factor input, expected output and unexpected output, and *V*_*m*_ and *V*_*g*_ represent the quantity of DMU under the common frontier and group frontier. *μ* and *θ* are intensity variables at these two levels.

In addition, according to the inclusion relationship of heterogeneity: *β*^*meta*^≥*β*^*group*^, group heterogeneity is caused by ‖*BD*‖. According to Hu et al. [57], the optimal production potential GMP is defined as the ratio of the potential (minimum) input required to achieve the optimal technical efficiency under the framework of agricultural multi-factor production. That is:

$$GMP=1-(1-\beta^{m})=\beta^{m}$$ (6)

The technology gap ratio (*TGR*) under the common frontier framework is equal to the ratio of the common frontier efficiency to the group frontier efficiency, that is:

$$TGR=\frac{1-\beta^{m}}{1-\beta^{g}}$$ (7)

The value is between [0,1] and reflects the gap between the group frontier and the common frontier technology level. The larger the *TGR*, the closer the actual production technology is to the potential production technology level; The smaller the *TGR*, the farther the actual production technology is from the potential technical level.

### 3.3. Metafrontier Malmquist-Luenberger index model

In this paper, the total factor productivity of green agriculture is measured. In order to reflect the research field, Metafrontier Malmquist Luenberger is recorded as GATFP (total factor productivity of green agriculture).

The traditional Malmquist-Luenbeger exponent has no solution to linear programming, and does not have transitivity and additionality [58]. Pastor and Lovell [59] pointed out that Metafrontier Malmquist-Luenberger can effectively solve the problem that ML exponential linear programming is not feasible. Oh [60] applies the Metafrontier method to the Global Malmquist model and constructs the Metafrontier Malmquist-Luenberger exponential model. The highlight is that the production of all periods may be gathered and enveloped to build the global frontier, that is, *P*^*G*^(*x*) = *P*^1^(*x*^1^)∪*P*^2^(*x*^2^)∪⋯∪*P*^*T*^(*x*^*T*^), and the model is:

$$P^{G}(x)=\{\begin{array}{l} (y^{t},b^{t}):\sum_{t=1}^{T}\sum_{i=1}^{I}Z_{i}y_{im}^{t}\geq y_{m}^{t};\\ \sum_{t=1}^{T}\sum_{i=1}^{I}Z_{i}b_{in}^{t}\geq b_{n}^{t};\\ \sum_{t=1}^{T}\sum_{i=1}^{I}Z_{i}x_{ik}^{t}\geq x_{k}^{t} \end{array}\}$$ (8)

The Metafrontier Malmquist-Luenberger index consists of the distance between the two adjacent production points and the common frontier. At the same time, the model has transitivity, that is:

$$mGATFP_{t-1}^{t}=\sqrt{\frac{1-{\vec{D}}_{t-1}^{m}(x^{t},y^{t},b^{t};y^{t},-b^{t})}{1-{\vec{D}}_{t-1}^{m}(x^{t-1},y^{t-1},b^{t-1};y^{t-1},-b^{t-1})}\times \frac{1-{\vec{D}}_{t}^{m}(x^{t},y^{t},b^{t};y^{t},-b^{t})}{1-{\vec{D}}_{t}^{m}(x^{t-1},y^{t-1},b^{t-1};y^{t-1},-b^{t-1})}}$$ (9)

$$gGATFP_{t-1}^{t}=\sqrt{\frac{1-{\vec{D}}_{t-1}^{g}(x^{t},y^{t},b^{t};y^{t},-b^{t})}{1-{\vec{D}}_{t-1}^{g}(x^{t-1},y^{t-1},b^{t-1};y^{t-1},-b^{t-1})}\times \frac{1-{\vec{D}}_{t}^{g}(x^{t},y^{t},b^{t};y^{t},-b^{t})}{1-{\vec{D}}_{t}^{g}(x^{t-1},y^{t-1},b^{t-1};y^{t-1},-b^{t-1})}}$$ (10)

$mGATFP_{t-1}^{t}$ and $gGATFP_{t-1}^{t}$, which represent the total factor productivity level of green agriculture at the common frontier and the group frontier respectively. Its value is greater than 1, indicating that the total factor productivity of green agriculture has increased. According to Wang et al. [61], GATFP can be decomposed into:

$$\begin{array}{l} GATFP_{t-1}^{t}=\sqrt{\frac{1-{\vec{D}}_{t-1}(x^{t},y^{t},b^{t};y^{t},-b^{t})}{1-{\vec{D}}_{t-1}(x^{t-1},y^{t-1},b^{t-1};y^{t-1},-b^{t-1})}\times \frac{1-{\vec{D}}_{t}(x^{t},y^{t},b^{t};y^{t},-b^{t})}{1-{\vec{D}}_{t}(x^{t-1},y^{t-1},b^{t-1};y^{t-1},-b^{t-1})}}\\ =\sqrt{\frac{1-{\vec{D}}_{t-1}(x^{t-1},y^{t-1},b^{t-1};y^{t-1},-b^{t-1})}{1-{\vec{D}}_{t}(x^{t-1},y^{t-1},b^{t-1};y^{t-1},-b^{t-1})}\times \frac{1-{\vec{D}}_{t-1}(x^{t},y^{t},b^{t};y^{t},-b^{t})}{1-{\vec{D}}_{t}(x^{t},y^{t},b^{t};y^{t},-b^{t})}}\times \frac{1-{\vec{D}}_{t}(x^{t},y^{t},b^{t};y^{t},-b^{t})}{1-{\vec{D}}_{t-1}(x^{t-1},y^{t-1},b^{t-1};y^{t-1},-b^{t-1})}\\ =GATC_{t-1}^{t}\times GAEC_{t-1}^{t} \end{array}$$ (11)

GATFP index greater than 1 indicates that the total factor productivity of green agriculture increases, and less than 1 indicates that the total factor productivity of green agriculture decreases. Technological progress index (GATC) and technological efficiency index (GAEC) are greater than 1, which indicate technological progress and efficiency improvement, and less than 1, which indicate technological retrogression and efficiency decline, both reflect the technological changes, regional efficiency level in management, system, scale economy and so on [62]. Through the GATFP index and its decomposition items, we can analyze the trend of total factor productivity of green agriculture, and provide an improvement plan for the development of green agricultural economy according to the heterogeneity of the three regions.

## 4. Data and descriptive statistics

### 4.1. Index selection

This paper uses the panel data of 30 provinces in China except Hong Kong, Macao, Taiwan and Tibet from 1997 to 2019 for empirical analysis. Considering the availability and continuity of the data of each province and index, this paper selects the data after 1997 as the research period. According to the standard of Jun et al. [63] and Wang et al. [64], the whole country is divided into eastern, central and western regions.

The following are the agricultural input and output indicators that need to be clarified to calculate the total factor productivity of green agriculture:

According to Xavier [65], agricultural input is defined as land, labor, mechanical power and fertilizer input. In this paper, agricultural sown area is used as land input, because it reflects a series of agricultural planting activities such as transplanting crops. The employment of the primary industry reflects the actual utilization of labor force in a certain period of time, so it is regarded as labor input. At the same time, in this paper, the total power of agricultural machinery is expressed by mechanical input, and the fertilizer application is converted into pure amount (including nitrogen fertilizer, phosphorus fertilizer, potassium fertilizer and compound fertilizer used in agricultural production) is expressed by chemical input. The above indicators are derived from *China Statistical Yearbook*, *China Rural Statistical Yearbook and Provincial Statistical Yearbook (1998–2020)*, and individual missing values are filled by interpolation.

For agricultural output, it can be divided into expected output and unexpected output. In this paper, the gross domestic product of the primary industry is regarded as the expected output of agriculture, which can accurately reflect the real output level by eliminating the "intermediate consumption".

In this paper, agricultural carbon emissions and non-point source emissions are included in the research framework of non-expected output.

For carbon emissions, we use Wang and Lin [49] and Yang et al. [50] to calculate CO_2_ emissions according to the guidance method of IPCC, and calculate agricultural carbon emissions. IPCC’s method is a global measurement method, which avoids the error caused by different calculation caliber, so this paper uses IPCC’s method to calculate China’s agricultural carbon emissions. IPCC’s method gives the carbon source and emission coefficient of agricultural production, and its calculation model is as follows:

$$D_{t}=\sum C_{tj}\times F_{j}$$ (12)

*D*_*t*_ represents the total carbon emissions in the *t* year, *C*_*tj*_ represents the consumption of energy of category *j* in the *t* year, *F*_*j*_ is the carbon emission coefficient of energy of type *j*, where *j* = 1,2,3,4,5,6 represent the corresponding carbon sources, that is, the amount of chemical fertilizer application, the amount of pesticide use, the amount of agricultural film use, the area of cultivated land irrigation, the amount of agricultural diesel oil use and the area of cultivated land.

For the discharge of non-point source pollutants, according to *the Bulletin of the First National Pollution Source Census*, non-point source pollutants are defined as chemical oxygen demand (CODcr), total nitrogen (TN) and total phosphorus (TP), and the relationship between agricultural activities and pollutants is established according to Lai et al. [66] and Chen et al. [67]’s "Top-down" unit analysis method. The calculation formula is as follows:

$$ANSP=\sum EU_{activity}=\sum \sum EU_{classification}=\sum \sum \sum EU_{unit}\times EUA$$ (13)

In the formula, *ANSP* represents the sum of the emissions of agricultural non-point source pollution, that is, *CODcr*, *TN* and *TP*, *EU*_*activity*_ represents the activities that produce non-point source pollution, *EU*_*classification*_ represents the specific category of non-point source pollution, *EU*_*unit*_ is a specific unit that produces non-point source pollution, in which the activity is composed of categories, the category is composed of units, and *EUA* represents the pollutant emissions of a single unit. The calculation formula is as follows:

$$\begin{array}{l} EUA=\sum_{i}EU_{i}\rho_{ij}(1-\eta_{i})C_{ij}(EU_{ij},S)\\ =\sum_{i}PE_{ij}\rho_{ij}(1-\eta_{i})C_{ij}(EU_{ij},S) \end{array}$$ (14)

In the formula, *EU*_*i*_ representing the *i* unit, *ρ*_*ij*_ is the pollution intensity coefficient of the *i* unit pollutant *j*, *η*_*i*_ is the resource utilization efficiency correlation coefficient, *PE*_*ij*_ is the production amount of the pollutant *j*, *C*_*ij*_ represents the *j* emission coefficient of the *i* unit of pollutants, determined by *EU*_*ij*_, and the spatial characteristics *S*.

The survey indicators of non-point source pollution production units come from *China Rural Statistical Yearbook*, *China Statistical Yearbook* and Provincial Statistical Yearbook. The parameters such as pollution intensity coefficient and emission coefficient mainly come from the provincial data of *the first national pollution source census*: the relevant resource utilization efficiency coefficient, loss coefficient and other data refer to the treatment method of Zou et al. [68].

### 4.2. Analysis of data significance

After calculating the green agricultural total factor productivity according to the above indicators, this paper makes a single factor analysis of variance on the data of 30 provinces from 1998 to 2019. As shown in Table 1, the measured values have not only temporal significance, but also spatial significance.

**Table 1:** Result of a single factor analysis of variance.

Difference	SS	MS	F	P-value	F crit
			Time		
Inter-group	0.195756	0.009322	4.540282	1.51E-10	1.572417
Within-group	1.309887	0.002053			
Summary	1.505643				
			Space		
Inter-group	0.019209	0.000915	8.794417	9.47E-10	1.800885
Within-group	0.004576	0.000104			
Summary	0.023785				

## 5. Results and discussion

In this paper, the total factor productivity of green agriculture (GATFP) in 30 provinces of China from 1997 to 2019 is calculated and decomposed, and the factors affecting the total factor productivity of green agriculture are analyzed. Based on this, the regional difference and influence mechanism of total factor productivity of green agriculture are studied.

### 5.1. The development characteristics of the total factor productivity of green agriculture in China

**Fig 2:**
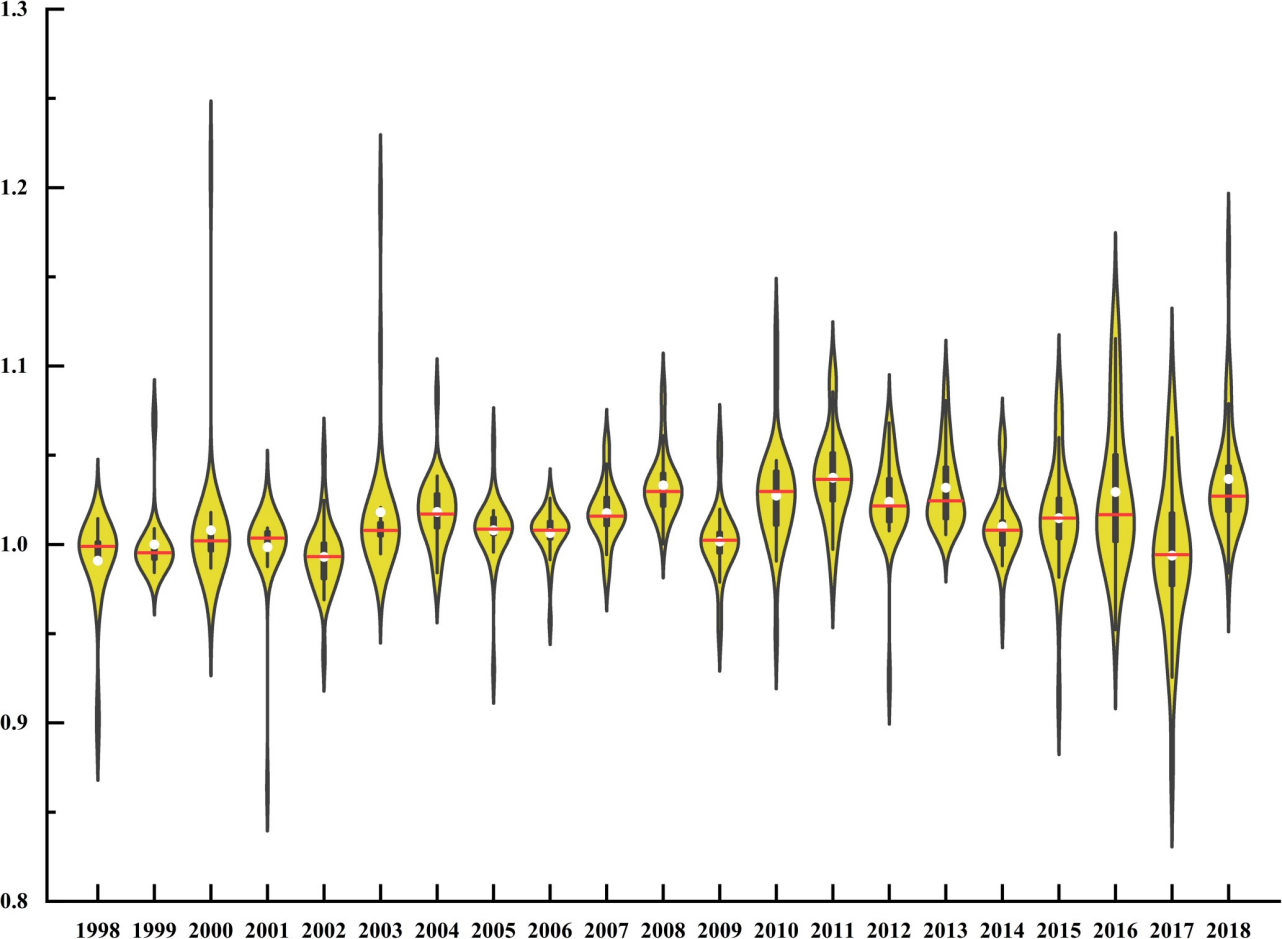
Variation of GATFP in China from 1998 to 2019.

Fig 2 is the change trend of total factor productivity of green agriculture in China during the study period, and the shape of the box in each year is determined by the productivity value of each province in that year. The longer the box, the more scattered the distribution of each province in that year, and the shorter the box, the more concentrated the distribution of each province. From this, we can see that GATFP shows the characteristics of first concentration and then dispersion in time. Before 2010, due to the great differences in economic level and social development between regions, growth poles will first be formed in all parts of the country, and then the economic development of surrounding areas will be improved through "diffusion effect", showing the concentrated characteristics of GATFP. However, in recent years, due to the rapid development of scientific and technological innovation capability, it is very likely that production relations cannot keep up with the development of productive forces in underdeveloped areas, thus showing the characteristics of "Matthew Effect".

**Fig 3:**
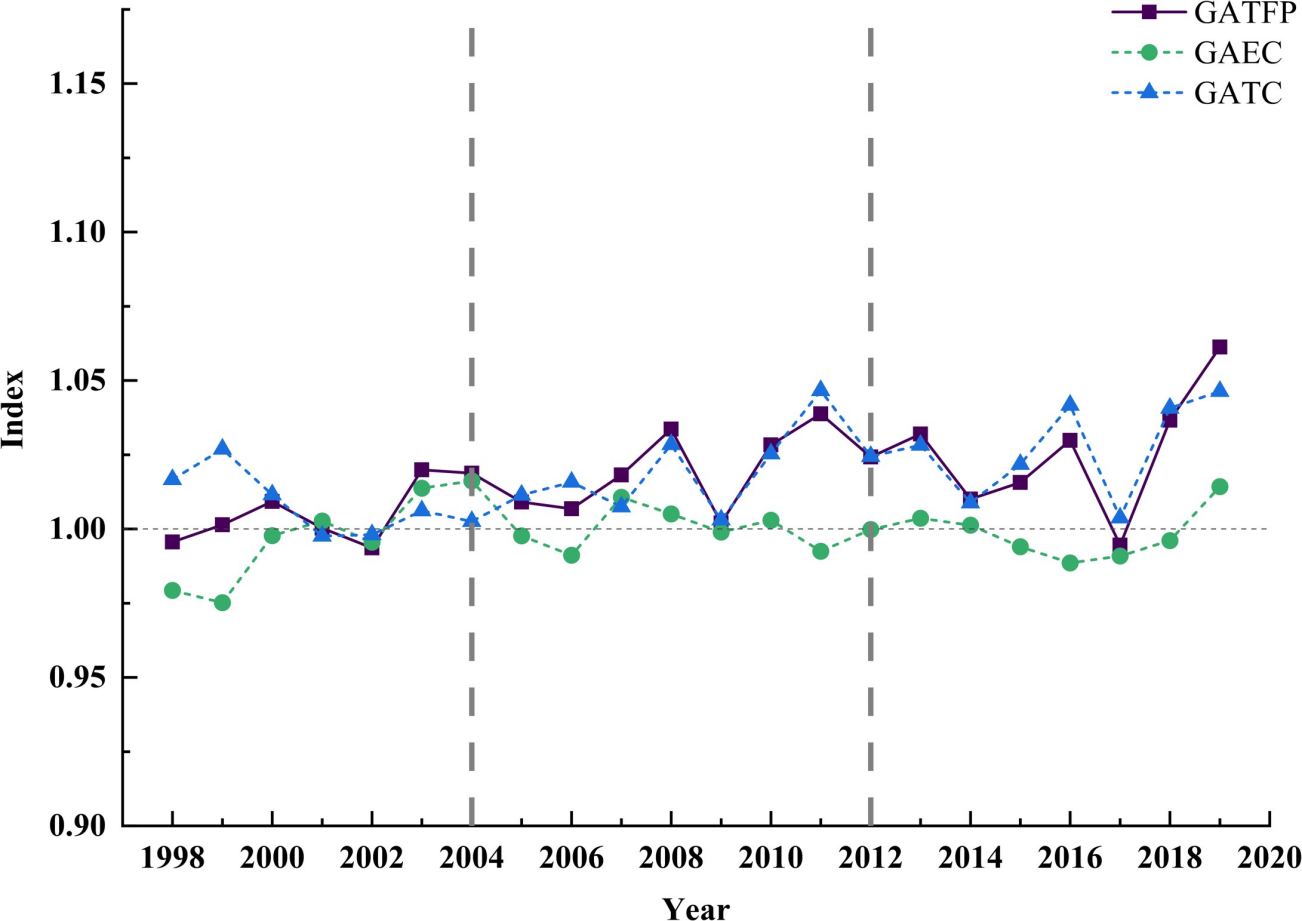
Variation of GATFP and its decomposition terms from 1998 to 2019.

Fig 3 is the trend of GATFP and its decomposition technology efficiency change (GAEC) and technology progress level (GATC). As can be seen from the figure, except for 1998 (0.9956), 2002 (0.9936) and 2017 (0.9946), the average of other years is more than 1, showing an upward trend over time, but the overall level is low. From 1997 to 2019, the total factor productivity index of green agriculture was 1.0173, that is, the average annual growth rate was 1.73%, and the cumulative average productivity index was 1.4533. That is to say, the cumulative growth rate during the study period (1997 = 1.0000) was 45.33%, and the cumulative growth was relatively fast.

From the national level, technological progress (1.0188) has a catalytic effect on GATFP, while technological efficiency (0.9985) has an inhibitory effect on GATFP. Technological progress is the core driving force of total factor productivity of green agriculture, the main reasons are: First, the support of agricultural science and technology investment and the improvement of production technology directly promote technological progress; Second, due to the inadequate use of agricultural production technology, the idle technical equipment and the limited large-scale development of agriculture. It has a weak inhibitory effect on GATFP because of low technical efficiency.

The volatility of GATFP over time is mainly affected by the changes of agricultural policy and economic policy. Gong [69] divides agricultural policy into three stages: 1994–1998, 1998–2004 and after 2004. Then Huang [70] expanded 2012–2017 to the fourth stage, and the research period of this paper is 1997–2019. According to the above criteria, it can be divided into three stages: 1997–2004, 2004–2012 and 2012–2019, as shown in Fig 3.

In the first stage, GATFP showed an integral upward trend. Due to the extraordinary natural disasters in China in 1998, agricultural production was seriously affected, so that the value of this point was less than 1, and then it rebounded in 1999. In 2001, China joined the World Trade Organization, and agricultural products can be freely circulated internationally. In the same year, the government abolished the quota procurement policy and reduced the protection measures for agricultural products. In order to cope with the challenges and competitions brought about by globalization, China’s technological progress in the field of agriculture has been accelerating, thus achieving the growth of GATFP. During this period, technological progress (1.0085) stimulated GATFP, and technological efficiency (0.9972) inhibited GATFP, and that effect of both are not obvious.

The second stage showed greater volatility, agricultural reform documents were issued intensively, and policy instability affected agricultural production. Among them, the abolition of agricultural tax in 2006 stimulated agricultural production and increased GATFP, followed by the global financial crisis in 2018, which made the prices of agricultural products continue to decline. It has a greater impact on agricultural production and reduced the growth rate in 2009. Subsequently, the economy recovered and the government increased subsidies for agricultural products, which led to a rebound in GATFP. During this period, the distribution of technological progress (1.0203) and technological efficiency (0.9998) stimulated and inhibited GATFP.

In the third stage, GATFP fluctuated slightly, but it achieved rapid development. During the 19th National Congress of the Communist Party of China, the government put forward the development strategy of "Rural Revitalization", stipulating that the contract period of land should be extended for another 30 years, which raised farmers’ awareness of land using rights, and would be more willing to increase agricultural investment by introducing advanced science and technology, thus making GATFP appear more. In 2017, due to frictions in trade activities, the import volume of agricultural products in the United States directly caused losses to farmers, resulting in total factor productivity less than 1. During this period, technological progress (1.0273) had a weak inhibitory effect on GATFP, while technological efficiency (0.9984) had a weak inhibitory effect.

In order to make GATFP develop better, we need to improve the technological level and innovation ability of the whole society, pursue efficient output and reduce environmental pollution. The government needs to increase investment in science, technology and environmental protection. In the special period, appropriate subsidies should be given to rural farmers to make agricultural production develop steadily. Only when technology, efficiency and policy play a positive role in all aspects, can the total factor productivity level of green agriculture in China develop continuously and healthily.

### 5.2. Temporal and spatial characteristics of total factor productivity of green agriculture in different regions

**Fig 4:**
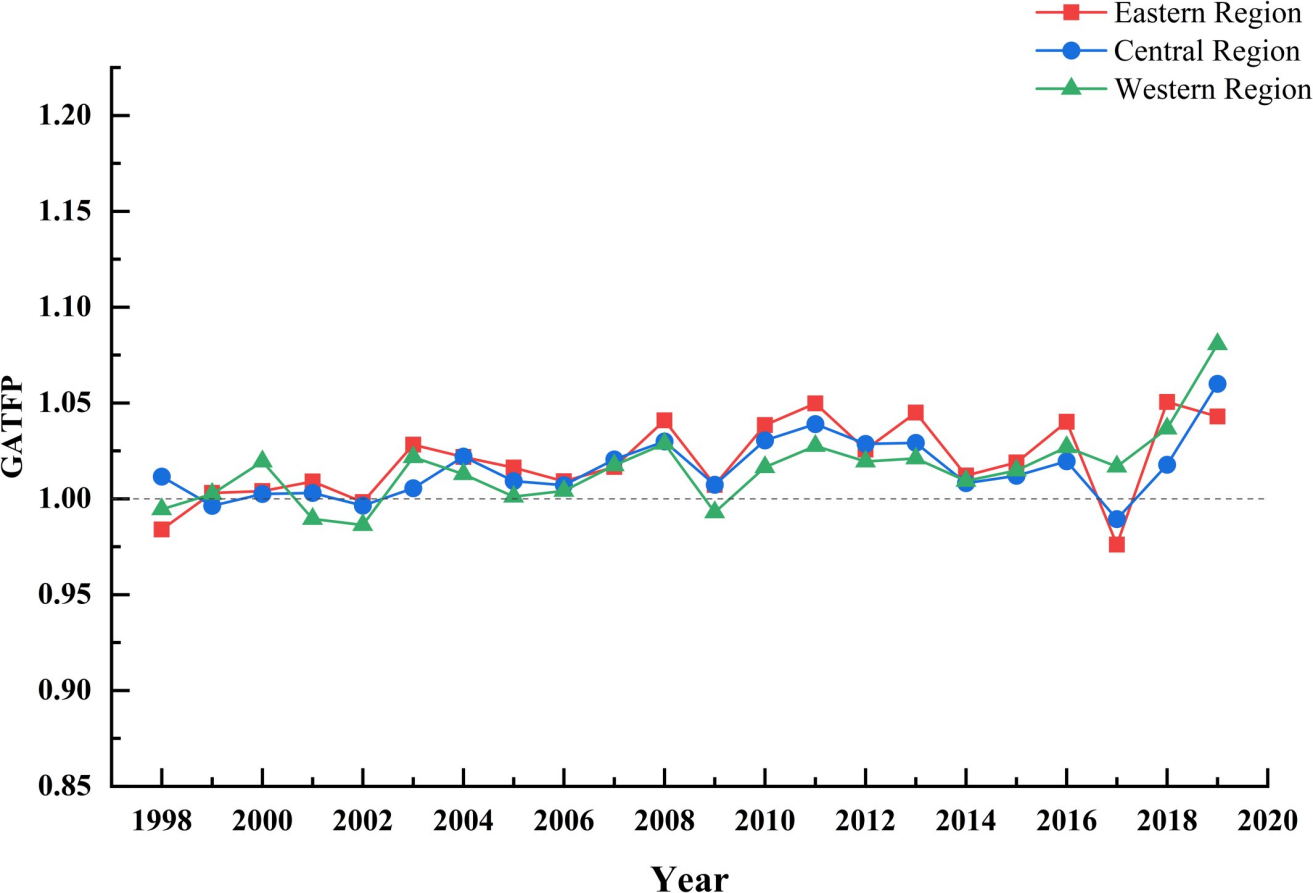
Changes of GATFP in the eastern, central and western regions from 1998 to 2019.

Fig 4 is the trend of GATFP over time in the eastern, central and western regions. From the time point of view, the time characteristics of the three regions are consistent with the overall trend of the whole country basically, showing the characteristics of fluctuation and rising, the GATFP index from high to low is eastern (1.0199), central (1.0157) and western (1.0155), the gap between regions is relatively small in most years.

**Fig 5:**
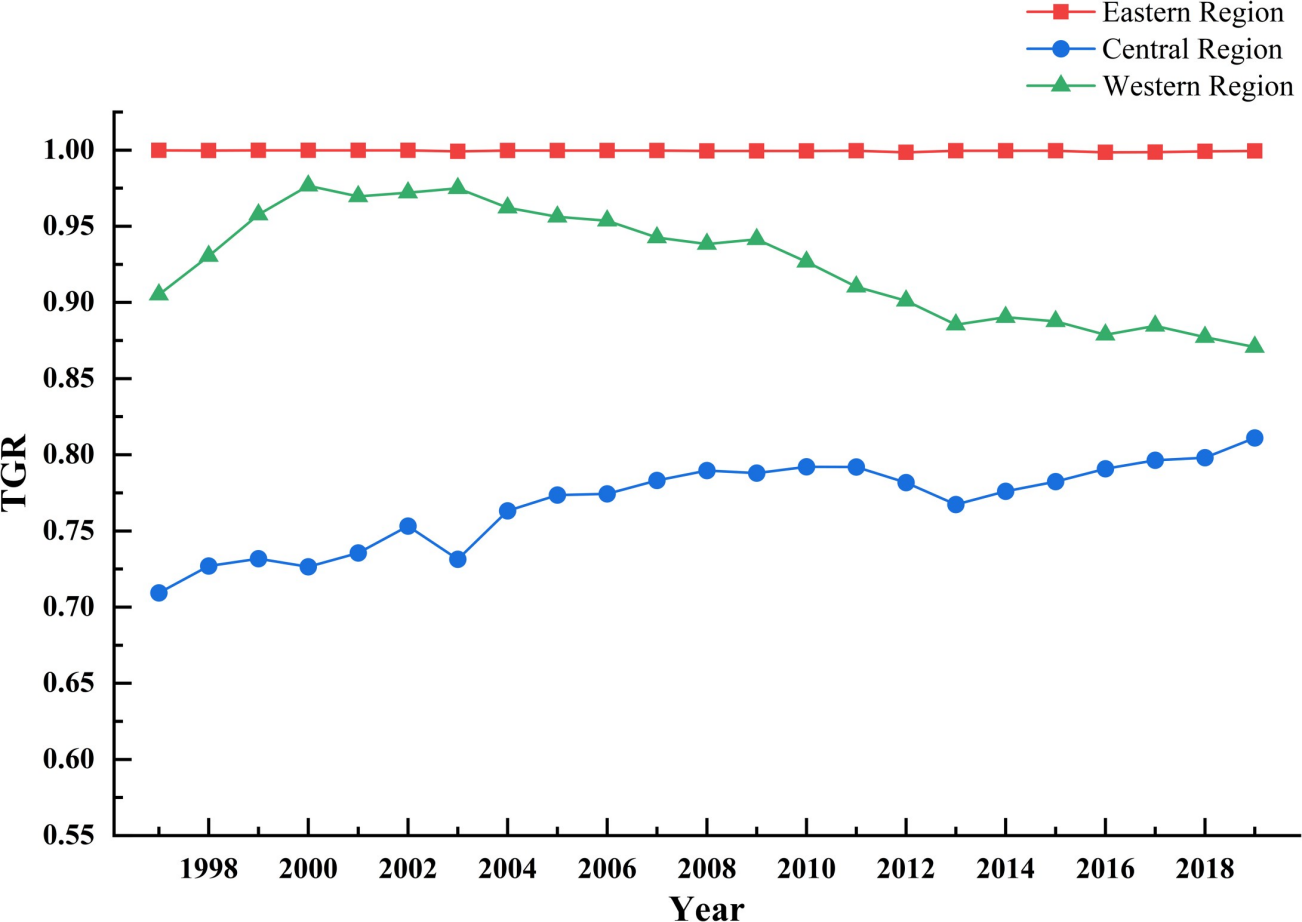
Changes in GMP in the eastern, central and western regions from 1997 to 2019.

Fig 5 is the trend chart of the production potential (GMP) of the three regions changing with time.

From the time point of view, the three regional changes have shown a downward trend gradually, before 2009 and after 2017, GMP declined in turn in the order of the middle, west and east, in the rest of the intermediate time, the total factor productivity of green agriculture in each group declined in turn in the west, east and middle. In the eastern, western and central regions, the annual average values are 0.7349, 0.6560 and 0.6439, that is, with the potential optimal production technology, the total factor productivity of green agriculture will reach 26.51%, 34.40%, 35.61%.

Compared with the eastern region, the central and western regions have greater production potential, which shows that the central and western regions need to fully absorb the technological advantages of economically developed areas and make production relations keep pace with the development of productive forces. At the same time, the government should strengthen guidance and support to keep up with the overall development of the whole country. Among them, the central region should give full play to its advantages in natural geological conditions, rationally apply technological innovation to agricultural production, improve its independent innovation ability, design a sustainable circular agricultural system, and reduce the waste of factor input and environmental pollution.

**Fig 6:**
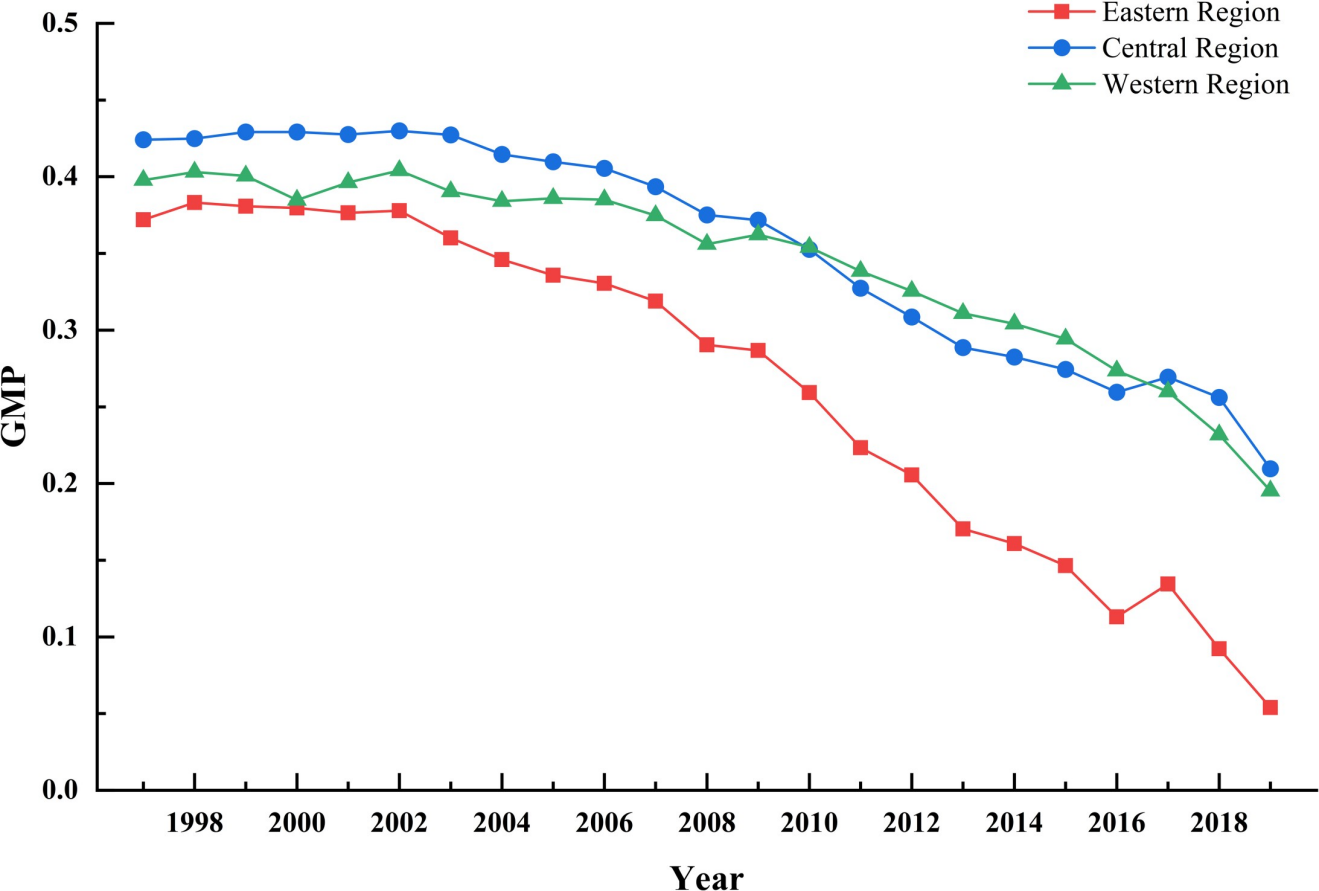
Changes in TGR in the eastern, central and western regions from 1997 to 2019.

Technology gap rate (TGR) reflects the gap between the productivity level of a specific group and the productivity level under the potential common frontier. The larger the value is, the closer the actual technical level of the decision making unit is to the optimal productivity technology level of the common frontier. Fig 6 reflects the trend of technology gap rate in the three regions over time.

From the time point of view, the eastern TGR is close to 1 during the study period, and the fluctuation range is small. The TGR in the western region is at a medium level and shows a downward trend, indicating that the level of technological innovation needs to be strengthened in the future to avoid the decline of TGR progress. TGR in the central region is at a low level, but it shows an upward trend, indicating that the situation in the central region has improved in recent years.

From the spatial point of view, the average TGR of the three regions is from high to low in the east (0.9995), the west (0.9258) and the middle (0.7684). The technical level of green agricultural productivity in most provinces and regions of the eastern and western groups is basically close to the optimal production technology level of the common frontier in the group, while the technical gap rate in the central part is small. The main reasons are the low efficiency of production technology and the large gap of production technology in the provinces of the region. Because of the ratio, improving the level of production technology and narrowing the technological gap between provinces are important ways to effectively improve the total productivity of regional green agriculture and to stabilize the development of total factor productivity of green agriculture in surrounding areas.

**Fig 7:**
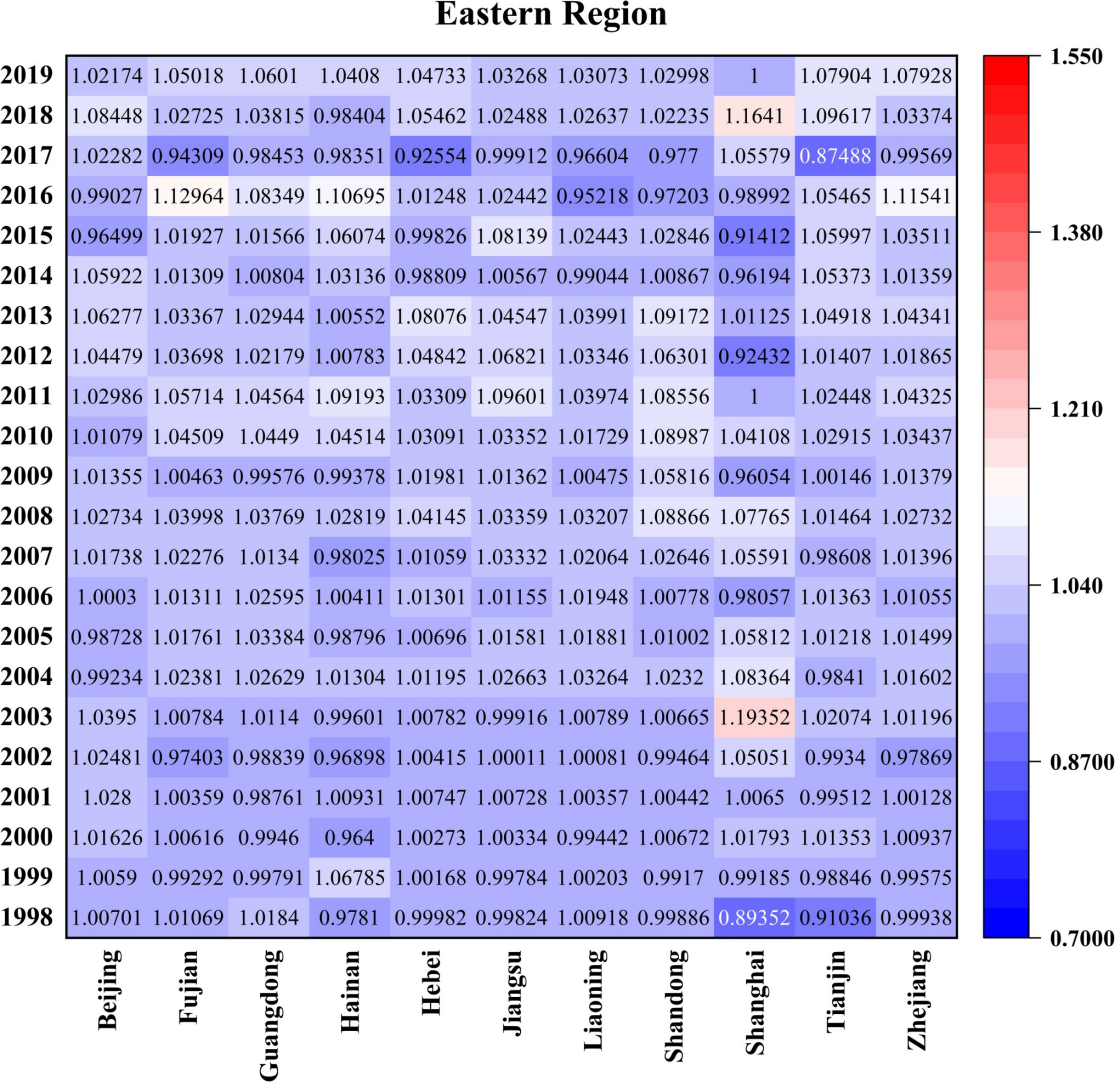
Time variation and average TGRs of GATFP in the eastern provinces.

**Fig 8:**
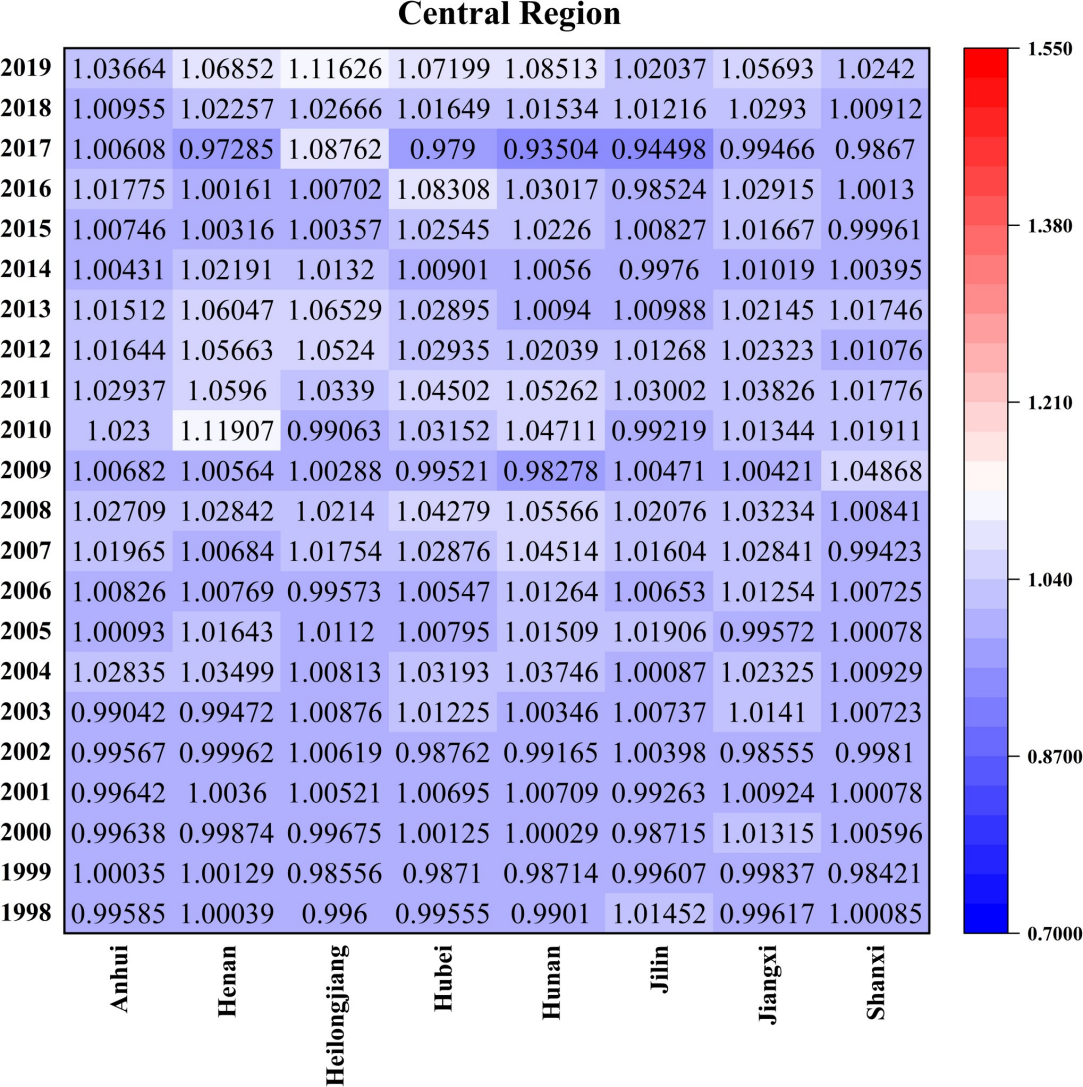
Time variation and average TGRs of GATFP in the central provinces.

**Fig 9:**
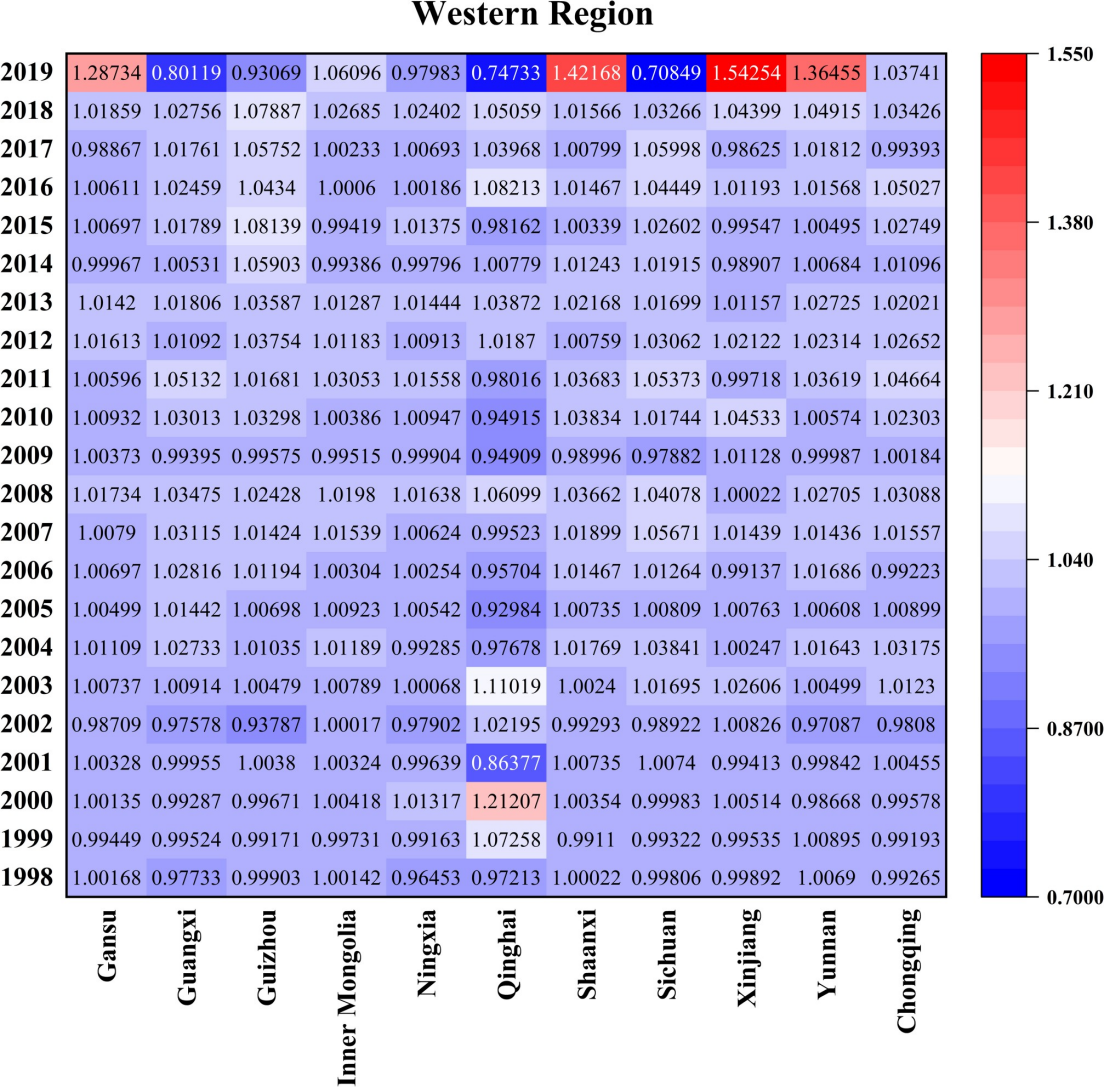
Time variation and average TGRs of GATFP in the western provinces.

**Fig 10:**
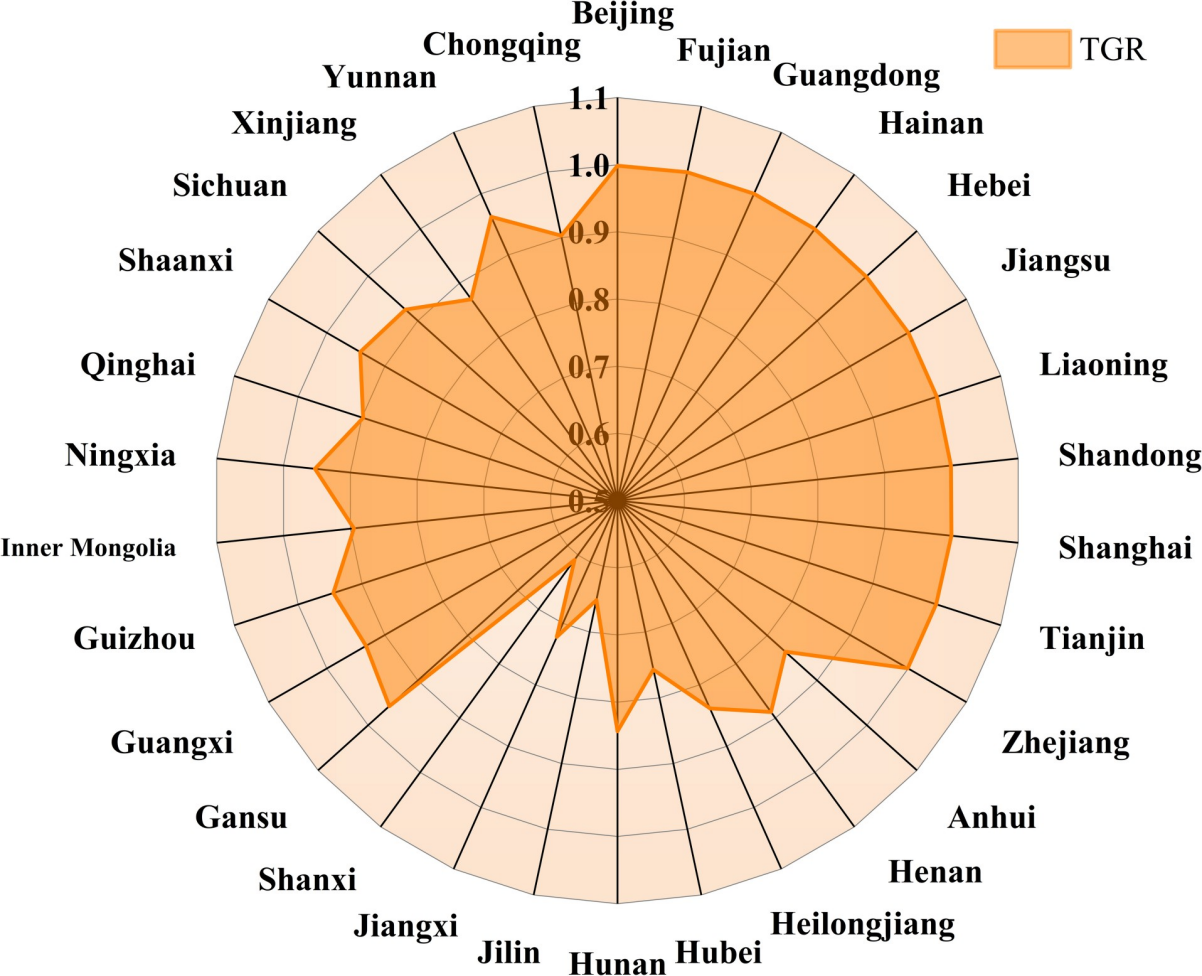
The average TGRs of GATFP in national provinces.

Figs 7–10 is the GATFP change and average technology gap rate of each province in different regions from 1997 to 2019. From the perspective of the three regions as a whole, the larger points of GATEP are concentrated around 2000 and after 2016, mainly because the tax reform has fully mobilized the enthusiasm of farmers and the rapid development of technological innovation ability in the new era.

From the perspective of specific provinces in different regions (Figs 7–10 and Table 2), The top five provinces in China are Guangdong (1.0000), Jiangsu (1.0000), Fujian (1.0000), Shanghai (0.9999) and Liaoning (0.9999) are all in the eastern region, while Shanxi (0.6093), Jilin (0.6516), Jiangxi (0.7220), Hubei (0.7574) and Anhui (0.8363) are all located in the central part.

**Table 2:** TGR ranking of some provinces.

No.	Comprehensive		No.	Eastern Region		Central Region		Western Region	
1	Guangdong	1.0000	1	Guangdong	1.0000	Henan	0.8888	Yunnan	0.9623
2	Jiangsu	1.0000	2	Jiangsu	1.0000	Hunan	0.8434	Gansu	0.9573
3	Fujian	1.0000	3	Fujian	1.0000	Heilongjiang	0.8388	Ningxia	0.9530
4	Shanghai	0.9999							
5	Liaoning	0.9999							
26	Anhui	0.8363							
27	Hubei	0.7574							
28	Jiangxi	0.7220	Bottom 3	Tianjin	0.9990	Jiangxi	0.7220	Qinghai	0.8981
29	Jilin	0.6516	Bottom 2	Beijing	0.9986	Jilin	0.6516	Inner Mongolia	0.8946
30	Shanxi	0.6093	Bottom 1	Hebei	0.9983	Shanxi	0.6093	Xinjiang	0.8705

The reasons can be explained from the total factor productivity level and technology gap of green agriculture: the overall GATFP value in the east is larger, and the core driving force of technological progress is larger. The closer it is to the optimal productivity technology level under the common frontier; GATFP in the western region is relatively average, indicating that the total factor productivity level of green agriculture in the region is relatively close, so there will not be some provinces to lower the average technology gap, so that the technology gap rate of the western region is in the middle level; The internal development of the central region was unbalanced in the early stage, and the situation has improved in recent years, so the average technology gap rate is at a low level due to the impact of the long-term imbalance in the early stage.

In order to narrow the gap of green agricultural economic development among regions, it is necessary to adjust the advantages and disadvantages of the technological efficiency, production potential and technological gap between the eastern, central and western regions. Farmers, enterprises, the government and other aspects should take various measures to comprehensively improve the total factor productivity level of green agriculture.

### 5.3. Effect of technology progress and technology efficiency in different region

**Fig 11:**
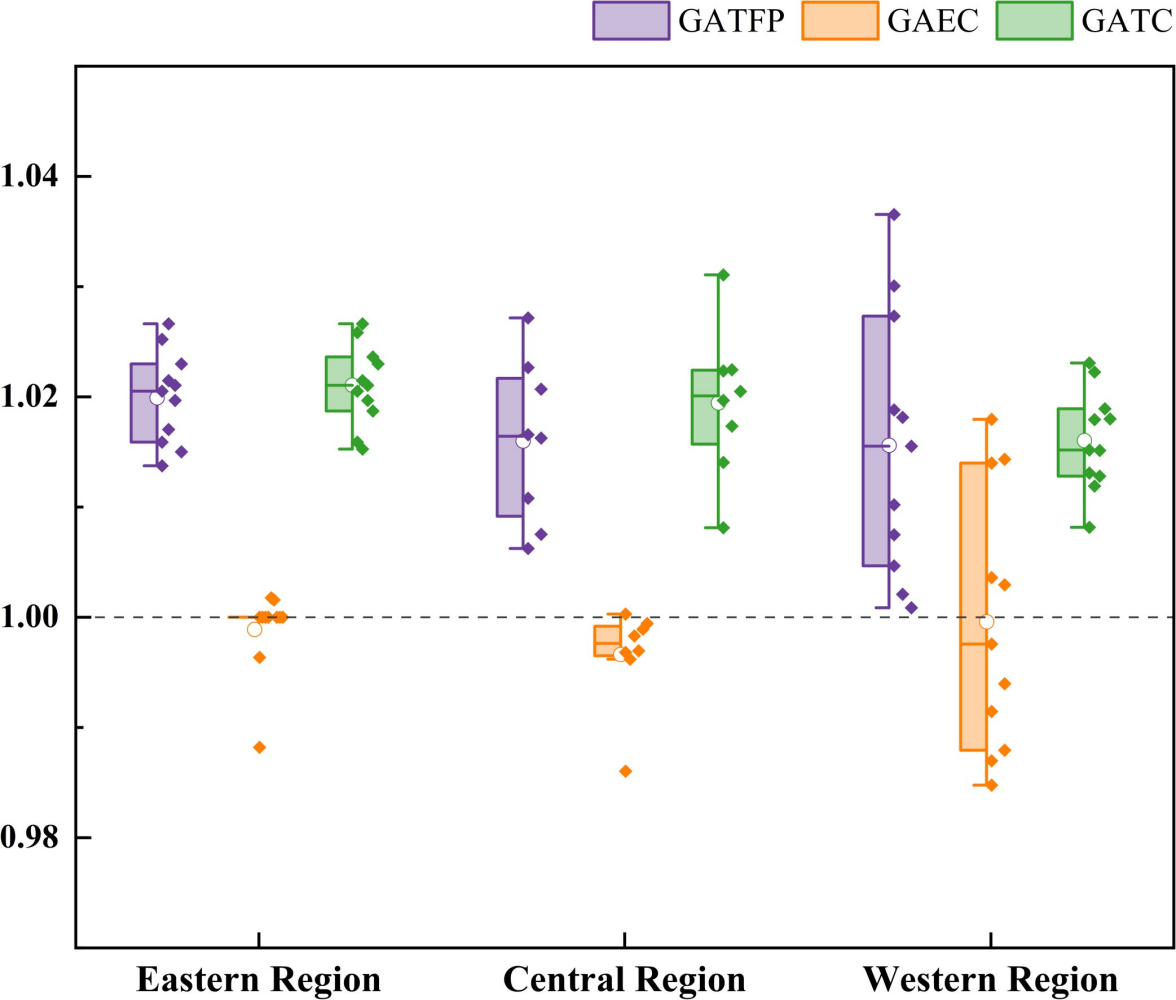
GATFP of the three regions and its decomposition.

Fig 11 is the semi-box chart of total factor productivity of green agriculture in the eastern, central and western regions, and each numerical point represents the average value of different provinces for many years.

As shown in Fig 11, the mean values of GATFP and its decomposition terms in the three regions are all greater than 1, and the mean values of technical efficiency are also greater than 1. The total factor productivity index of green agriculture is from high to low in the east (1.0199), middle (1.0160) and western (1.0156). Technological progress (1.0211 in the east, 1.0194 in the middle and 1.0160 in the west) is promoted, while technological efficiency (0.9989 in the east, 0.9966 in the middle and 0.9996 in the west) was consistent with the overall effect of the whole country.

**Fig 12:**
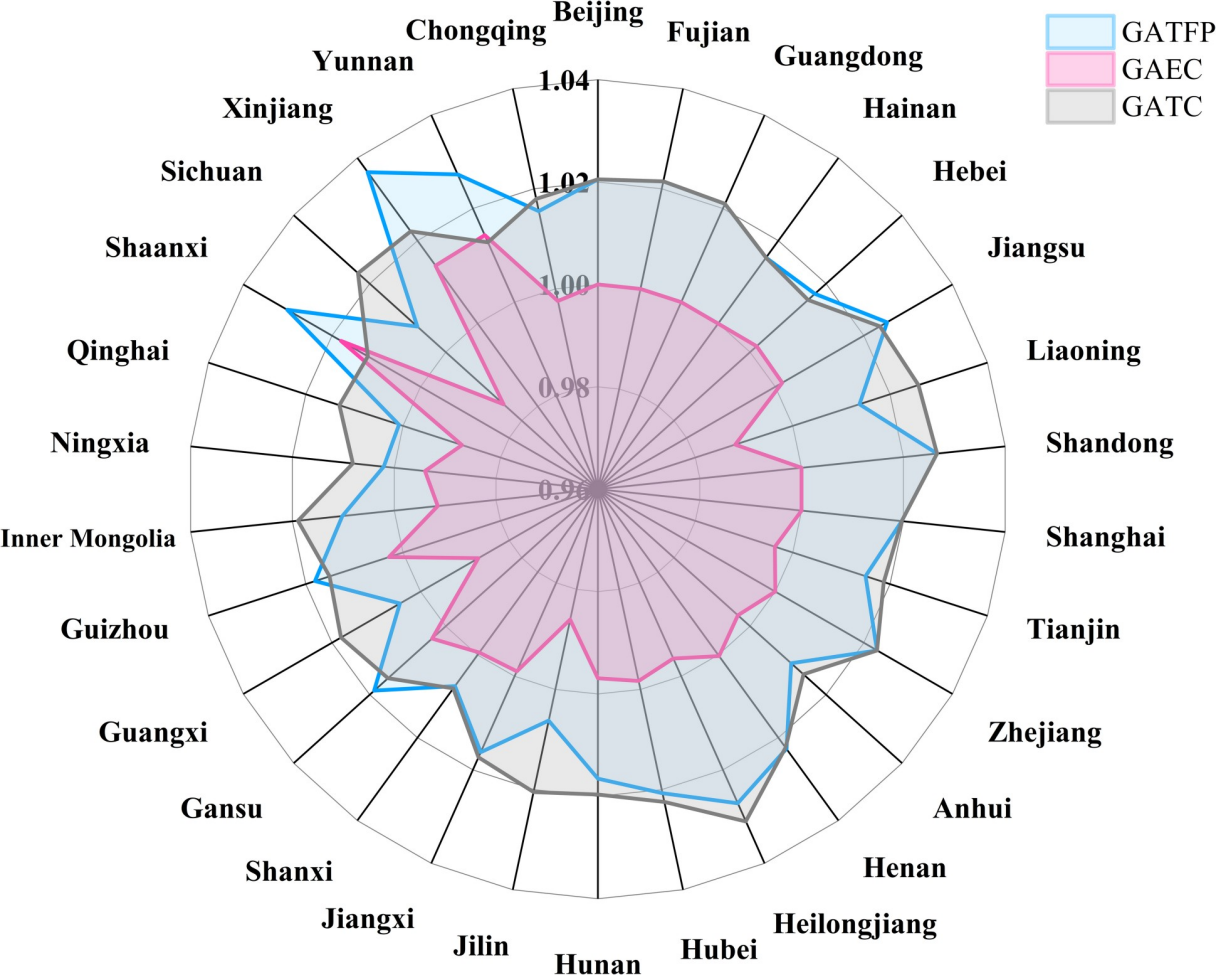
GATFP of national provinces and its decomposition.

According to the specific situation of each province (Fig 12 and Table 3), the top five provinces of China’s GATFP are Xinjiang (1.0365), Shaanxi (1.0301), Yunnan (1.0273), Heilongjiang (1.0272) and Shandong (1.0266), and these five provinces are in the top five of GAEC or GATC. The technical efficiency of Xinjiang plays a role in promoting total factor productivity, and the scale effect is strong. Shaanxi is located at the junction of the western and central regions, with high-quality natural geological conditions, suitable for large-scale agricultural production. And because the level of technological progress in Shaanxi is relatively high in the western region, GATFP is in the forefront.

**Table 3:** Top and bottom five rankings of GATFP, GAEC and GATC in China.

No.	GATFP		GAEC		GATC	
1	Xinjiang	1.0365	Shaanxi	1.0180	Heilongjiang	1.0311
2	Shaanxi	1.0301	Yunnan	1.0143	Shandong	1.0266
3	Yunnan	1.0273	Xinjiang	1.0140	Liaoning	1.0258
4	Heilongjiang	1.0272	Gansu	1.0036	Jiangsu	1.0236
5	Shandong	1.0266	Guizhou	1.0030	Sichuan	1.0231
26	Sichuan	1.0075	Liaoning	0.9882	Qinghai	1.0131
27	Jilin	1.0062	Qinghai	0.9879	Yunnan	1.0128
28	Guangxi	1.0047	Guangxi	0.9870	Shaanxi	1.0119
29	Ningxia	1.0021	Jilin	0.9860	Ningxia	1.0082
30	Qinghai	1.0009	Sichuan	0.9848	Shanxi	1.0081

Qinghai (1.0009), Ningxia (1.0021), Guangxi (1.0047) and Jilin (1.0062) and Sichuan (1.0075) ranked the last five in GATFP and are all underdeveloped areas of agricultural economy in the central and western regions. Because the level of technological progress in the western region is at a low level in the whole country, the conditions of agricultural production have not reached the national average level, so it is at the end of the ranking. In the ranking of the three indicators, the frequency of the eastern provincial cities is low, which shows that the economically developed areas are not necessarily good in technical efficiency. It is difficult to form a scale effect, which makes the effective technology investment insufficient and the technology efficiency inhibits the growth of GATFP. Moreover, according to the ranking of the three indicators, we can see that the development of the central and western provinces is quite different, and further key policy support is needed to narrow the regional gap.

In order to further develop the level of green total factor productivity in China’s agriculture, it is necessary to improve technological progress and efficiency at the same time, and their effects should not be ignored. First of all, enterprises need to improve their own technical level, develop and apply sustainable circulation systems, and expand output on the premise of reducing environmental pollution. At the same time, the government should strengthen policy guidance, encourage the introduction of advanced technology, narrow regional differences, adjust measures to local conditions according to the development of different regions, and devote itself to the development of total factor productivity of green agriculture from various aspects.

## 6. Conclusions and policy implications

This paper calculates and decomposes the green total factor productivity of China from 1997 to 2019, and the following conclusions are obtained:

Firstly, from the national level, China’s GATFP basically showed an upward trend, with rapid growth and obvious fluctuations over time. Technological progress has a promoting effect on GATFP, while technological efficiency has a weak inhibitory effect.

Secondly, from the differences of the three regions, we can see that GATFP decreases in the order of eastern, central and western regions. Moreover, the overall characteristics of the three regions over time are similar to the national trend. There are disadvantages in scale production in the eastern region, great production potential in the central region, and worrying prospects in the technological gap level in the western region, which have brought hidden dangers to the improvement of total factor productivity of regional green agriculture.

Thirdly, the effect of technological progress and technological efficiency on GATFP in three regions is similar to that of the whole country. The differences lie in the characteristics between the provinces within the regions. The eastern part has strong technical advantages, and the technical efficiency level of most areas is above the average level. The central and western region has a strong efficiency advantage, which is mainly reflected in the large-scale production and scale effect.

Based on the conclusion of this study, the following policy recommendations can be extended:

Firstly, it is advisable to pay more attention to regional differences, adapting measures to local conditions, utilizing agglomeration effect and diffusion effect, adjusting input and output, and increasing policy support. The country should continuously reduce environmental pollution, and comprehensively improve the agricultural green total factor productivity. In the process of implementation, it may face the problem of uneven distribution among regions, which has certain challenges for decision-making.

Secondly, for the eastern region, we can increase investment in scientific research, design and develop new production modes, and fully apply existing technologies to agricultural production. Improve production efficiency and reduce environmental pollution. For example, the comprehensive efficiency of agriculture in Liaoning Province is relatively high, but more than 50% of the areas fail to achieve economies of scale in agricultural production. By increasing the popularization of agricultural production technology, the agricultural efficiency of Liaoning has been greatly improved in recent years.

Thirdly, for the central region, we should give full play to its geographical advantages, absorb the diffusion effect of the growth pole in the eastern region, and fill in the greater production potential of the region. At the same time, we should pay attention to environmental protection, increase investment and control pollution in agricultural production. Although the implementation of this scheme can theoretically improve GATFP, it may make the production input and environmental protection input unbalanced in the central region, resulting in poor financial support.

Finally, for the western region, on the one hand, the government needs to increase support, comprehensively improve the level of regional science and technology, actively introduce advanced technology, and constantly adapt to the development of new productive forces. On the other hand, we need to apply technology to production activities reasonably and improve the agricultural development model accurately. In the process of effectively improving rural productivity, we may face the problem that farmers’ long-term production habits are difficult to change. So how to effectively improve the content of human capital in the western region is also a big challenge.

In a word, China should take various measures to coordinate regional development [71].
